# Supplementary material for: Metabolic reconstruction identifies strain-specific regulation of virulence in Toxoplasma gondii
Source: Mol Syst Biol. 2013 Nov 19;9:708. doi: 10.1038/msb.2013.62 (PMC4039375; doi:10.1038/msb.2013.62)
Supplement: Supplementary Information — Supplementary Figures S1–5 [file msb201362-s1.pdf]

SUPPLEMENTAL INFORMATION

TABLE OF CONTENTS

Page 1 Table of Contents

Page 2 Supplementary references

Page 3 List of Supplemental Tables

Page 4 Supplemental Figures 1-5

SUPPLEMENTARY REFERENCES

References Used to Define Essential Reactions for *Plasmodium falciparum*

Crowther GJ, Napuli AJ, Gilligan JH, Gagaring K, Borboa R, Francek C *et al* (2011). Identification of inhibitors for putative malaria drug targets among novel antimalarial compounds. *Mol Biochem Parasitol* **175:** 21-29.

Fatumo S, Plaimas K, Mallm J-P, Schramm G, Adebiyi E, Oswald M *et al* (2009). Estimating novel potential drug targets of Plasmodium falciparum by analysing the metabolic network of knock-out strains in silico. *Infection, Genetics and Evolution* **9:** 351-358.

Ginsburg H (2006). Progress in in silico functional genomics: the malaria Metabolic Pathways database. *TRENDS IN PARASITOLOGY* **22:** 238-240.

Preuss J, Hedrick M, Sergienko E, Pinkerton A, Mangravita-Novo A, Smith L *et al* (2012). High-throughput screening for small-molecule inhibitors of plasmodium falciparum glucose-6-phosphate dehydrogenase 6-phosphogluconolactonase. *J Biomol Screen* **17:** 738-751.

Yeh I, Hanekamp T, Tsoka S, Karp PD, Altman RB (2004). Computational analysis of Plasmodium falciparum metabolism: organizing genomic information to facilitate drug discovery. *Genome Res* **14:** 917-924.

LIST OF SUPPLEMENTAL TABLES

Supplemental Table I. Summary of *i*CS382. The metabolic model represented as a list of reactions ready to be imported into MATLAB using COBRA Toolbox. Enzymes associated with multiple reactions are differentiated by an alphabetical suffix to the EC number. Metabolites in each reaction are suffixed with compartment information, such as “[m]” for mitochondrion.

Supplemental Table II. List of enzymes with predicted gene mappings.

Supplemental Table III. Predicted impact of single enzyme knockouts on parasite growth.

Supplemental Table IV. Predicted impact of double enzyme knockouts on parasite growth.

Supplemental Table V. List of metabolites that map to KEGG chemical Ids listed in Supplemental Table 1

Supplemental Table VI. List of citations that map to PubMed Ids listed in Supplemental Table 1

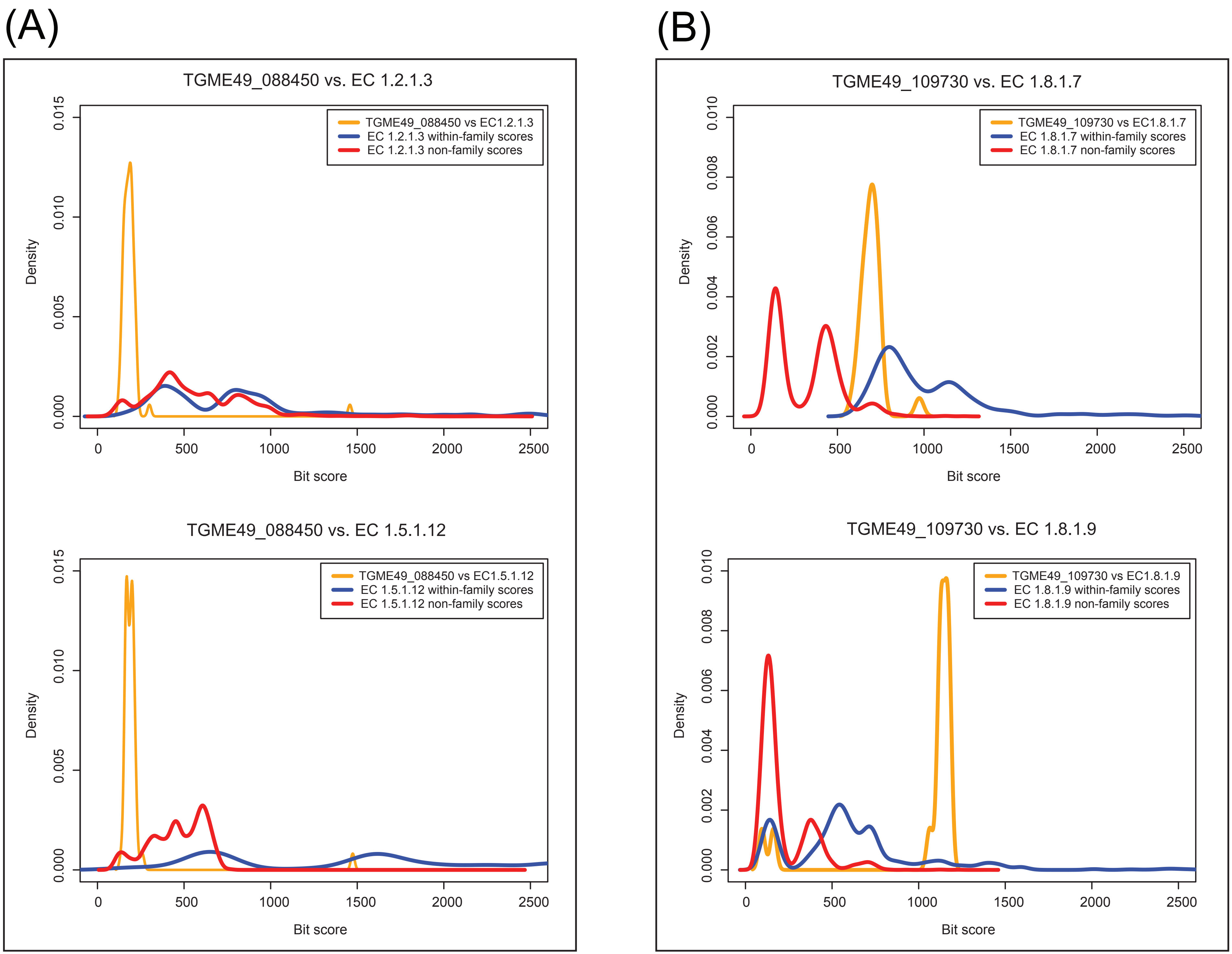

Supplemental Figure 1

Supplemental Figure 1. DETECT predictions for TGME49_088450 and TGME49_109730

(A) The distribution of scores for alignments of TGME49_088450 against EC 1.2.1.3 (top) and EC 1.5.1.12 (bottom) protein sequences compared to the respective within-family and non-family members alignments. The density distributions indicate that the alignment scores of TGME49_088450 correlate more closely with the positive alignment profile generated for EC 1.5.1.12 family members compared to EC 1.2.1.3 family members. Specifically, in the plot for EC 1.2.1.3, most hits (represented by the tall peak) are very low-scoring (close to having an almost non-significant bit alignment score) and are slightly biased to the negative hits. A minority of hits (small peak on the far right) overlap with a very small portion of 1.2.1.3 positive hits. However, given the majority of the positive hit distribution occurs around a bit score of ~400 and ~800, it is not surprising that EC 1.2.1.3 obtains a comparably lower probability score (implying that TGME49_088450 is less likely to belong to EC 1.2.1.3 enzymes). In the plot for EC 1.5.1.12, again, most hits are very low-scoring and biased towards negative hits; however, a minority (small peak on far right) overlap with a much larger section of the positive hit distribution (other large section of the positive hit distribution occurs around a bit score of ~600). This contributes to a higher probability score and suggests that TGME49_088450 is more likely to belong to the family of enzymes annotated as EC 1.5.1.12. (B) Similarly, alignment scores for TGME49_109730 correlate more closely with the positive alignment profile generated for EC 1.8.1.9 family members compared to EC 1.8.1.7 family members In the plot for EC 1.8.1.9, the majority of the hits to EC 1.8.1.9 enzymes (i.e. tall peak) are very high scoring (> 1000 bit score) and overlap with a region of the positive hit distribution for EC 1.8.1.9. The small peak in this plot is essentially uninformative since it corresponds to very low scoring hits and is ambiguous in its contribution to the positive or negative hit distribution. These aspects of the plot help to explain the higher probability score, and therefore greater likelihood that TGME49_109730 belongs to the family of enzymes annotated as EC 1.8.1.9. In the plot for EC 1.8.1.7, the majority of hits for TGME49_109730 (tall peak) are of relatively moderate/low score (~650 bit score) with some overlap to both positive and negative hit distributions, but with a slight bias towards the positive hit distribution. Together, the lack of very high-scoring hits (as is the case with EC 1.8.1.9) and overlap with both positive and negative hit distributions results in a lower probability score (less likely to belong to enzymes annotated as EC 1.8.1.7).

(A) Relative Expression RH : Me49

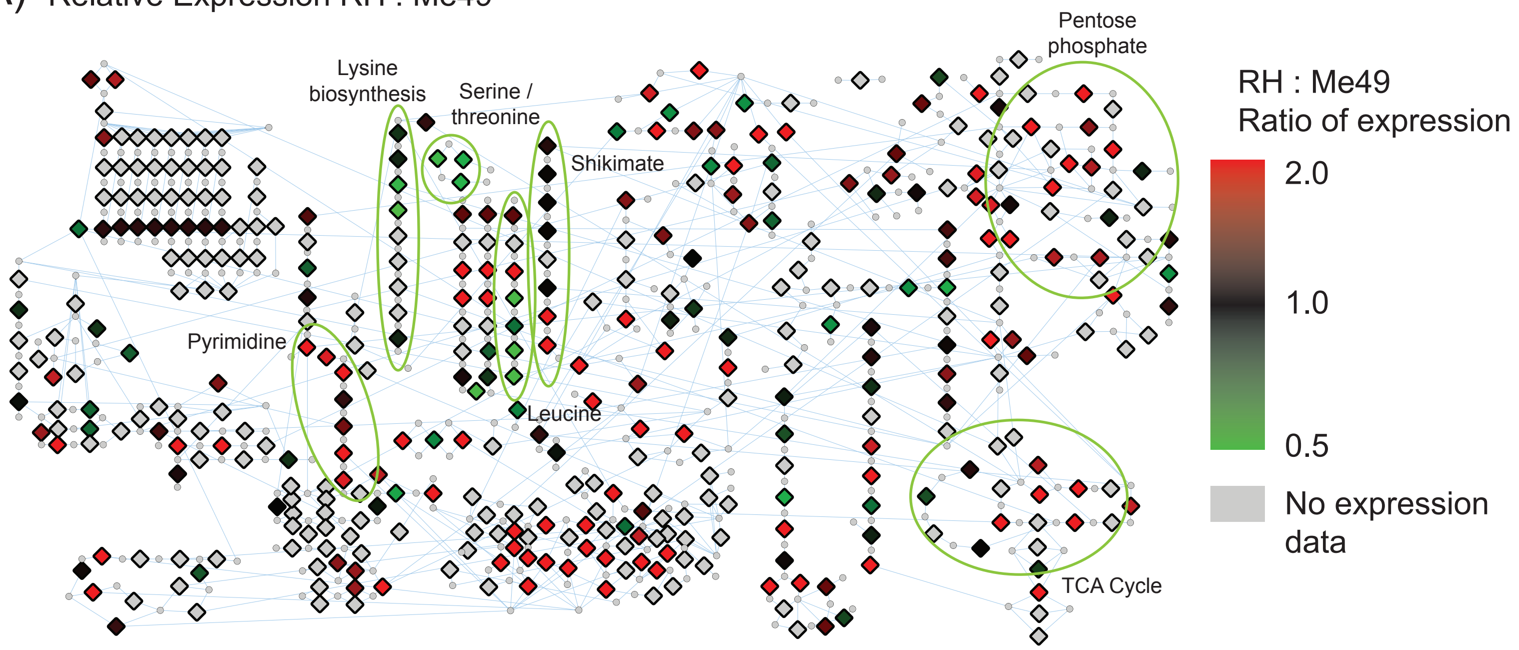

(B) Bottleneck Reactions

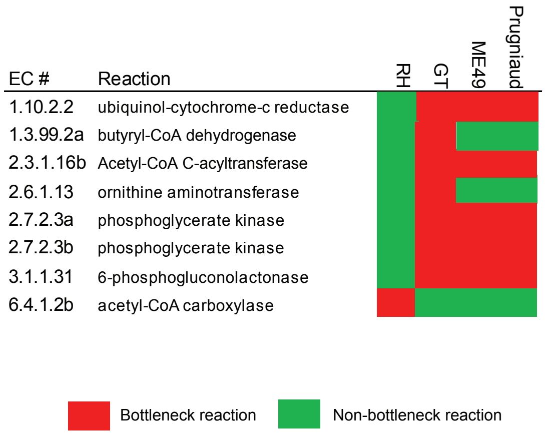

Supplemental Figure 2. Strain and species differences in *T. gondii* metabolism
(A) Relative expression of enzymes mapped onto the metabolic reconstruction comparing the two strains: RH and Me49. (B) Bottleneck reactions defined for the four strains: RH, GT, Me49 and Prugniaud. (C) Network visualization of reactions predicted and/or validated to be essential in *T. gondii* and *P. falciparum*.

(C) Diversity of essential enzymes - *Plasmodium falciparum : Toxoplasma gondii*

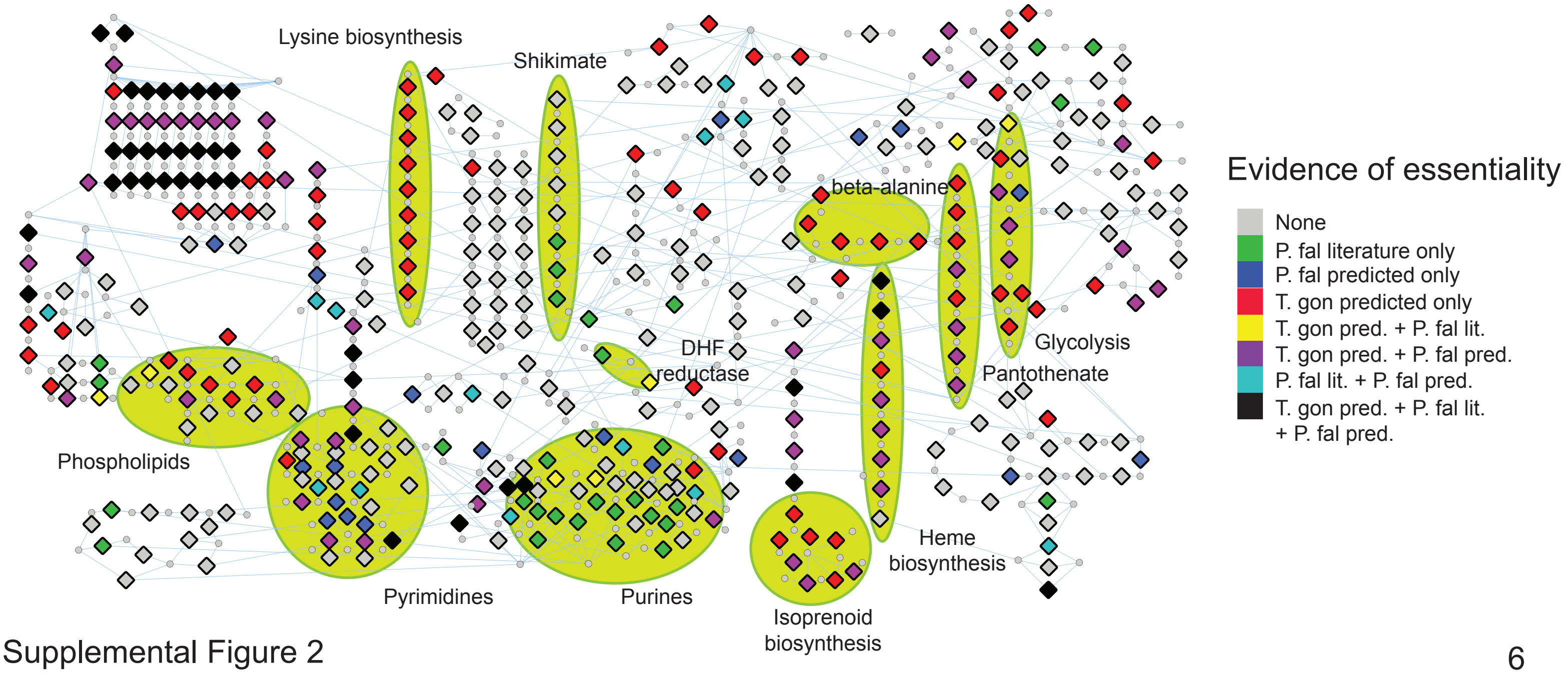

Supplemental Figure 2

(A)

Ubiquinol-cytochrome-c reductase (1.10.2.2)

		To			
		NoEffect	Minor	Major	Essential
From	NoEffect	192	1	0	0
	Minor	21	0	0	0
	Major	10	0	5	0
	Essential	0	5	1	165

Phosphoglycerate kinase in the cytosol (EC:2.7.2.3a)

		To			
		NoEffect	Minor	Major	Essential
From	NoEffect	193	0	0	0
	Minor	19	2	0	0
	Major	0	3	12	0
	Essential	0	0	2	169

Phosphoglycerate kinase in the apicoplast (EC:2.7.2.3b)

		To			
		NoEffect	Minor	Major	Essential
From	NoEffect	193	0	0	0
	Minor	16	2	3	0
	Major	0	4	11	0
	Essential	0	0	4	167

6-phosphogluconolactonase (EC:3.1.1.31)

		To			
		NoEffect	Minor	Major	Essential
From	NoEffect	193	0	0	0
	Minor	19	2	0	0
	Major	3	0	12	0
	Essential	0	0	3	168

(B)

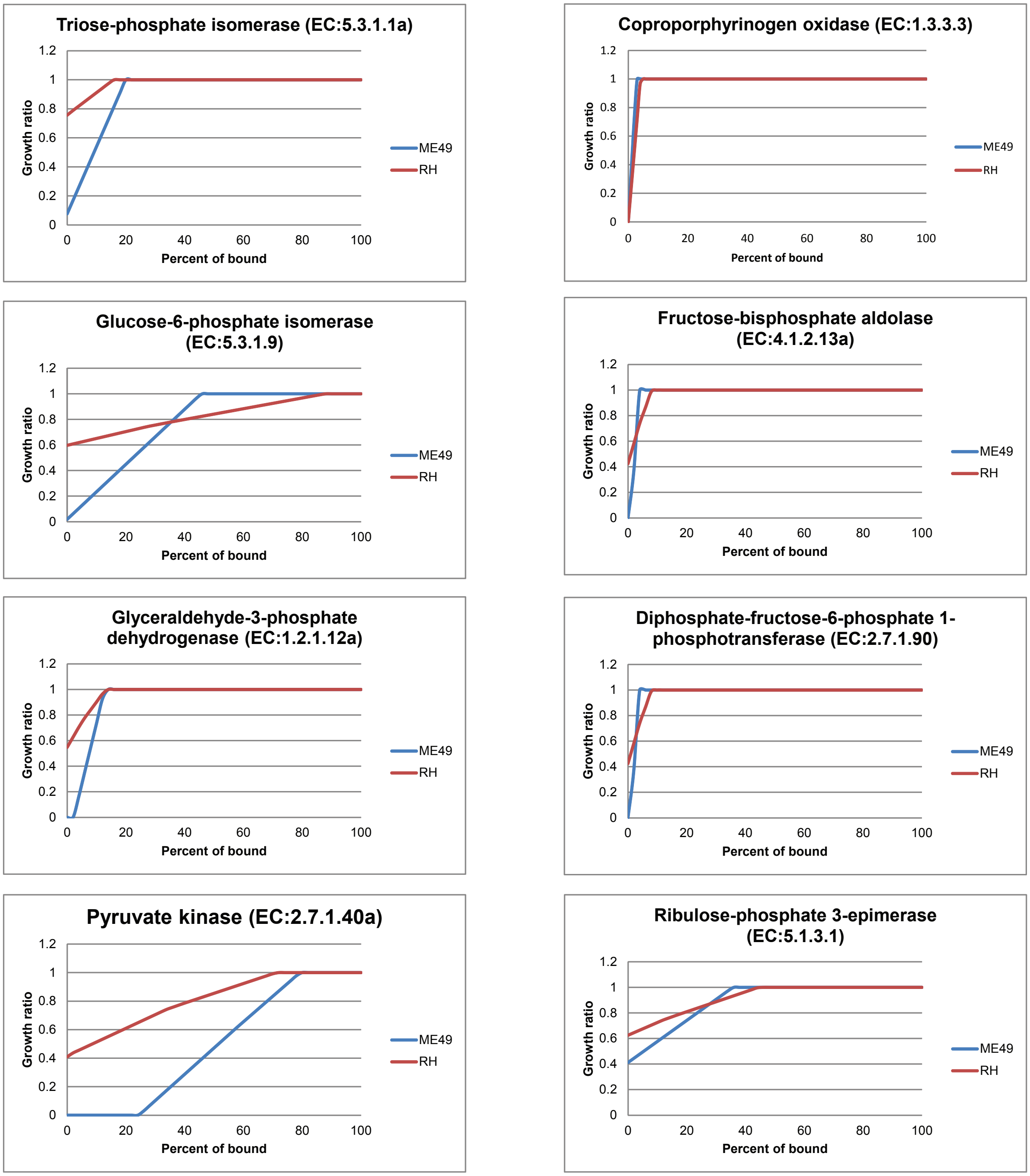

Supplemental Figure 3

Supplemental Figure 3. Sensitivity analyses exploring the impact of altering flux constraints on individual reactions

(A) Changes in growth rates predicted for single reaction knockouts for the four reactions identifying as impacting category assignment for the knockouts upon altering their upper constraint to the maximum allowing in the Me49 model. Most changes in category assignment are relatively subtle, e.g. from a gene knockout predicted to have a ‘minor effect’ (80-99% growth rate) to having no effect. (B) Effect of lowering the constraint for eight selected reactions, from 100-1% of the assigned constraint in the RH and Me49 models. Note for each reaction, the decrease in growth rate with respect to decreasing constraint results in a shallower gradient for strain RH compared to strain Me49, consistent with one of the main findings of our study that strain Me49 is predicted to be more sensitive to inhibition of enzymes involved in energy production pathways. Also note, the constraints applied in both models for these reactions are not limiting growth rate.

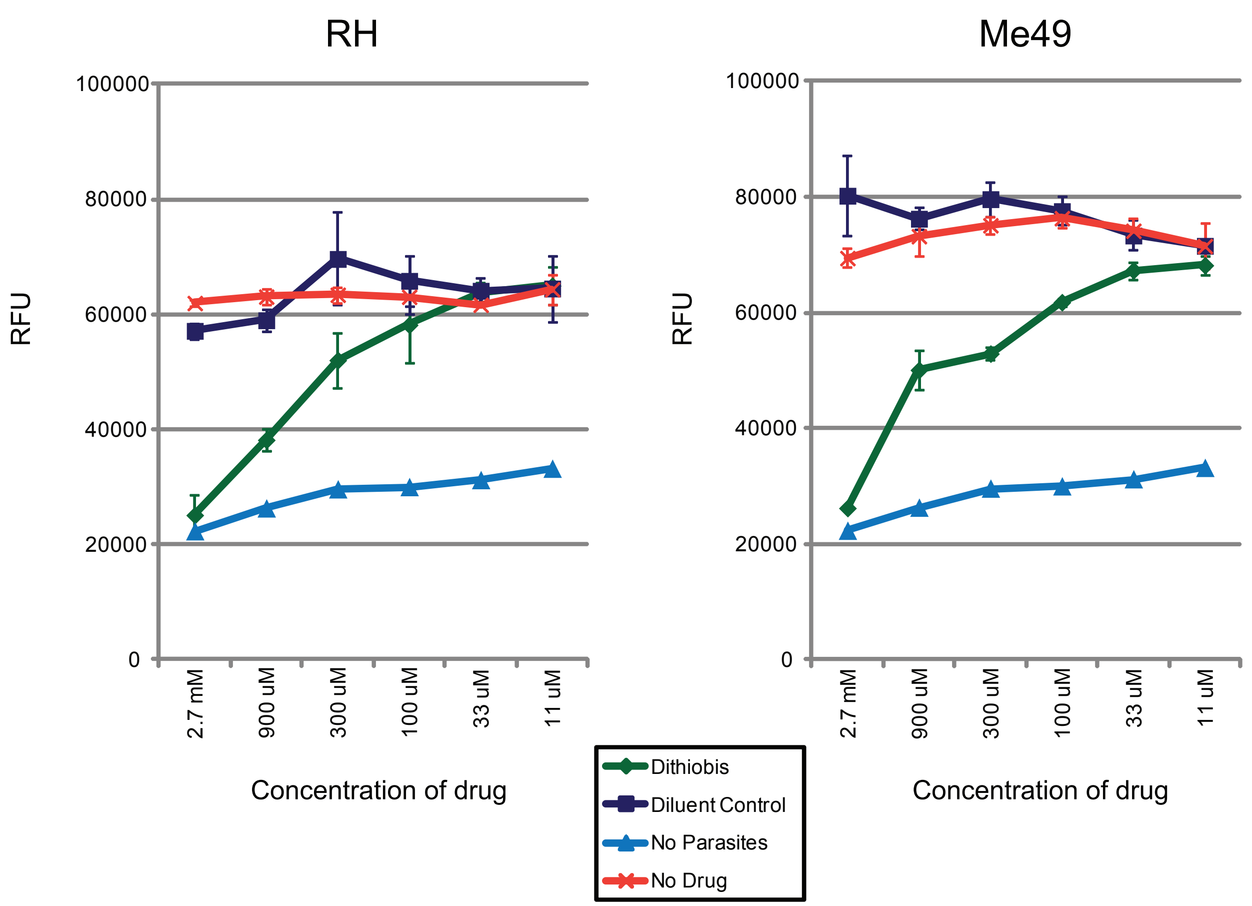

Supplemental Figure 4. Growth assays of Toxoplasma strains under treatment with dithiobis
7-day growth assays for Type I (RH) and Type II (Me49) strains under treatment with dithiobis targeting coproporphyrinogen oxidase (EC 1.3.3.3).

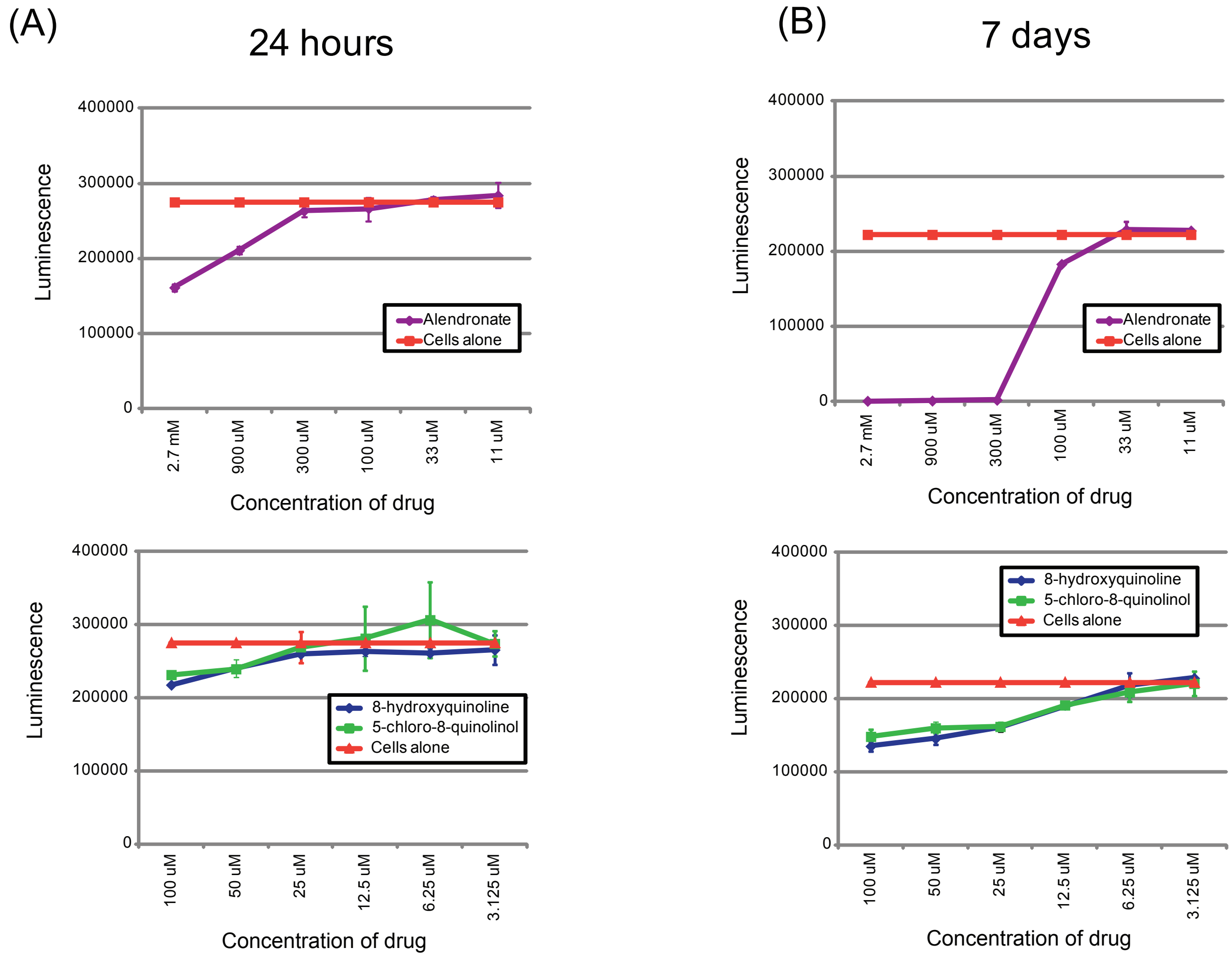

Supplemental Figure 5. Host cell viability assays for three compounds targeting two enzymes operating in glycolysis.
(A) Viability of host cells after 24 hours of exposure to the compounds: alendronate, 8-hydroxyquinoline and 5-chloro-8-quinolinol. (B) Viability of host cells after 7 days of exposure to the compounds: alendronate, 8-hydroxyquinoline and 5-chloro-8-quinolinol.
